# 7-Hydroxyflavone Alleviates Myocardial Ischemia/Reperfusion Injury in Rats by Regulating Inflammation

**DOI:** 10.3390/molecules27175371

**Published:** 2022-08-23

**Authors:** Qunhui Zhang, Yanfeng Peng, Jiangyu Liu, Yongjing Yang, Zhangjie Hu, Yi Zhou, Jing Ma, Dejun Zhang

**Affiliations:** 1 Research Center for High Altitude Medicine, Key Laboratory of High-Altitude Medicine (Ministry of Education), Key Laboratory of Application and Foundation for High Altitude Medicine Research in Qinghai Province (Qinghai-Utah Joint Research Key Lab for High Altitude Medicine), Qinghai University, Xining 810001, China; 2 College of Eco-Environmental Engineering, Qinghai University, Xining 810016, China

**Keywords:** 7-Hydroxyflavone, myocardial ischemia/reperfusion injury, p38, ERK1/2, JNK, NF-κB, cardioprotection

## Abstract

Inflammation is the primary pathological process of myocardial ischemia/reperfusion injury (MI/RI). 7-Hydroxyflavone (HF), a natural flavonoid with a variety of bioactivities, plays a crucial role in various biological processes. However, its cardioprotective effects and the underlying mechanisms of MI/RI have not been investigated. This study aimed to explore whether pretreatment with HF could attenuate MI/RI-induced inflammation in rats and investigate its potential mechanisms. The results showed that pretreatment with HF could significantly improve the anatomic data and electrocardiograph parameters, reduce the myocardial infarct size, decrease markers of myocardial injury (aspartate transaminase, creatine kinase, lactate dehydrogenase, and cardiac troponin I), inhibit inflammatory cytokines (IL-1β, IL-6, and TNF-α), suppress oxidative stress, and recover the architecture of the cardiomyocytes. The cardioprotective effect of HF was connected with the regulation of the MAPK/NF-κB signaling pathway. What is more, molecular docking was carried out to prove that HF could be stably combined with p38, ERK1/2, JNK, and NF-κB. In summary, this is a novel study demonstrating the cardioprotective effects of HF against MI/RI in vivo. Consequently, these results demonstrate that HF can be considered a promising potential therapy for MI/RI.

## 1. Introduction

Ischemic heart disease (IHD) is an epidemic and catastrophic disease, which jeopardizes human health and is becoming the primary cause of death worldwide [1]. The restoration of reperfusion in the cardiomyocytes in a timely and effective manner can markedly reduce the extent of myocardial infarction and restore the cardiac function, and even improve the patient prognosis [2]. However, it can also lead to myocardial damage and dysfunction, which is called myocardial ischemia/reperfusion injury (MI/RI) [3]. It is estimated that the incidence of MI/RI exceeds 30% and directly leads to adverse cardiovascular events. The pathophysiological processes of MI/RI are complex and include inflammation, oxidative stress, apoptosis, etc. [4]. In addition, the detailed mechanism of MI/RI is unclear. Anti-MI/RI strategies and the development of more effective cardioprotective strategies are the focuses of drug development [5]. Consequently, it is increasingly imperative to search for drugs for the treatment of MI/RI.

The accumulating evidence demonstrates that MI/RI causes myocardial oxidative stress and inflammation, which could eventually trigger apoptosis in the cardiomyocytes [6]. Mitogen-activated protein kinase (MAPK)/nuclear factor-κB (NF-κB) signaling pathways are involved in the above process of MI/RI [7,8,9]. Inflammation might be a significant factor in the whole pathophysiological progression of MI/RI [10]. A large number of inflammatory cells penetrate the area of the lesion soon after ischemia. The release of interleukin-6 (IL-6), interleukin-1β (IL-1β), and tumor necrosis factor-α (TNF-α) aggravate the myocardial injury, ultimately leading to apoptosis [11,12,13]. What is more, the acute response of inflammation and the infiltration of the inflammatory cells in the cardiomyocytes exacerbates myocardial damage. The occurrence of oxidative stress-triggered apoptosis during MI/RI is an additional factor [14]. Thus, reducing the inflammatory response, oxidative stress, and apoptosis could protect the cardiomyocytes from MI/RI.

Among the traditional Tibetan medicines (TTMs), *Oxytropis falcata* Bunge (*O. falcata*) is a species of the genus *Oxytropis*, which is mainly located in Qinghai, Tibet, Gansu, etc. According to the Tibetan medical book *JingZhuBenCao*, *O. falcata* has the effects of astringence and hemostasis, and has been used for the treatment of inflammation in folk medicine [15]. Our previous studies demonstrated that the flavonoids of *O. falcata* have anti-inflammatory, antioxidant, and anti-MI/RI pharmacological effects [15]. 7-Hydroxyflavone (HF) is a kind of flavonoid that we isolated from *O. falcata* previously, which has a beneficial role in maintaining the viability of hypoxia/reoxygenation-induced H9c2 cardiomyocytes. HF has been proven to have anti-inflammatory and antioxidant effects [16,17]. However, there is no evidence that HF has a direct anti-MI/RI effect, and its mechanisms are still unclear.

**Figure 1:**
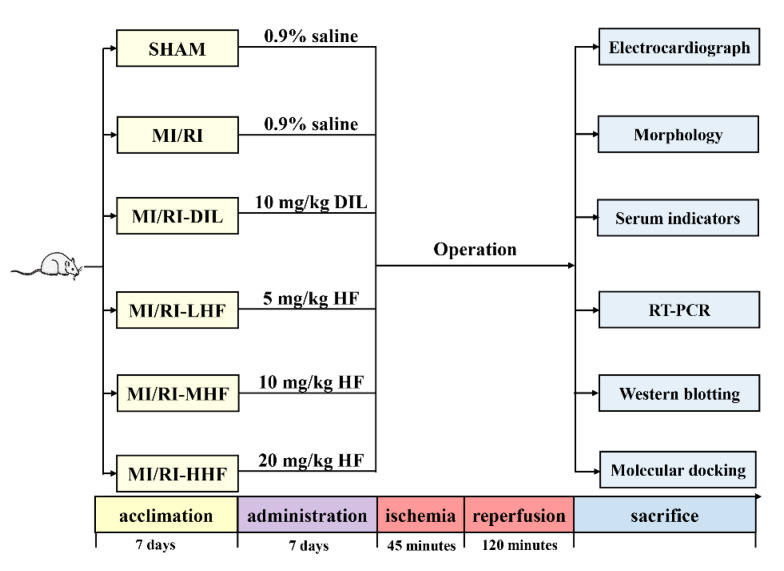
The design of this study.

Consequently, in this study, we first pretreated the rats with HF. Then, an MI/RI rat model was established by ligating the left anterior descending coronary artery (LAD) to investigate the effects of HF. Finally, quantitative real-time PCR (qRT-PCR), western blotting (WB), and molecular docking analysis methods were used to elucidate the underlying mechanism (Figure 1).

## 2. Results

### 2.1. HF Improves the Anatomic Data

To determine the effects of HF on the anatomical parameters, the ratio of heart weight to body weight (RHB), the ratio of liver weight to body weight (RLIB), the ratio of kidney weight to body weight (RKB), the ratio of spleen weight to body weight (RSB), the ratio of thymus weight to body weight (RTB), and the ratio of lung weight to body weight (RLUB) were used to assess the efficacy of HF. We found that the values of RHB, RLIB, RKB, RSB, and RTB in the MI/RI group were higher than those in the SHAM group (*p* < 0.05). Compared with the MI/RI group, the values of RHB, RLIB, RLUB, RSB, and RTB were lower in MI/RI-DIL, MI/RI-MHF, and MI/RI-HHF groups (*p* < 0.05). The anatomic data can be seen in detail in Table S1.

### 2.2. HF Improves the Electrocardiograph (ECG) Parameters

**Figure 2:**
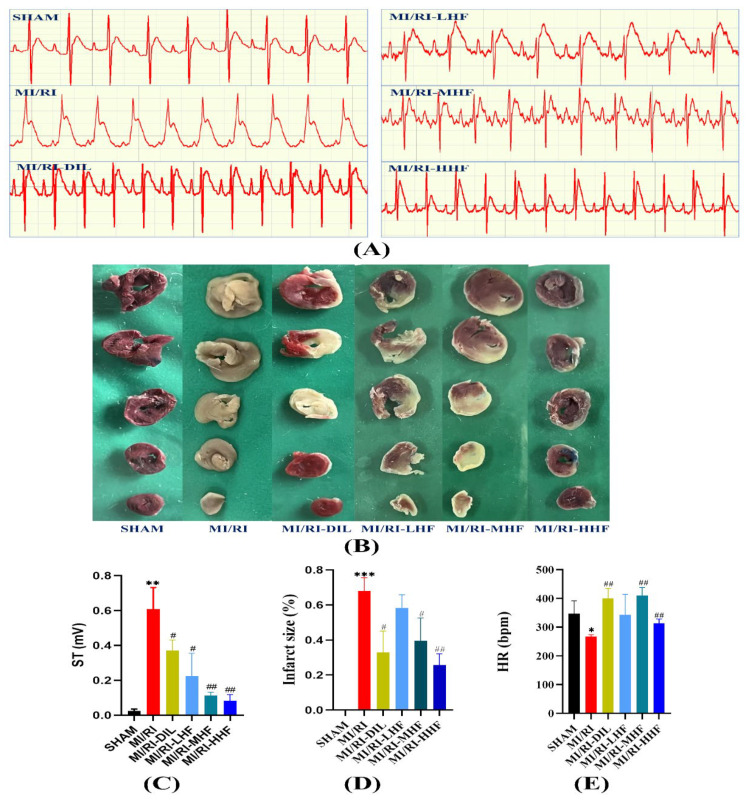
Effects of HF on the electrocardiograph (ECG) parameters and infarct size. (**A**) Representative ECGs of each group. (**B**) Representative images from each group in the TTC staining (*n* = 3). (**C**) The elevation of the ST segment from each group on the electrocardiograph (*n* = 3). (**D**) Quantitative analysis of the myocardial infarction area in each group (*n* = 3). € Heart rate from each group (*n* = 3). Results were expressed as mean ± SD. * *p* < 0.05, ** *p* < 0.01, *** *p* < 0.001 vs. SHAM group. ^#^ *p* < 0.05, ^##^ *p* < 0.01 vs. MI/RI group.

To investigate the effects of HF on the ECG, the ECG and HR were used to appraise the effects. Compared with the SHAM group, a significant elevation in the ST segment of the ECG and a significant reduction in the heart rate were observed in the MI/RI group (*p* < 0.05; Figure 2A,C,E). Compared with the MI/RI group, the pretreatment with DIL and HF significantly ameliorated these changes in the MI/RI-DIL, MI/RI-LHF, MI/RI-MHF, and MI/RI-HHF groups (*p* < 0.05; Figure 2A,C,E).

### 2.3. HF Reduces on IS in Rat MI/RI Model

To explore the effects of HF on the IS, TTC staining was used. The cardiac tissues were stained with 2% 2,3,5-triphenyltetrazolium chloride (TTC) while the infarct size (IS) was evaluated. Compared with the SHAM group, a significant growth in the IS was detected in the MI/RI group (*p* < 0.05; Figure 2B,D). Compared with the MI/RI group, the pretreatment with DIL and HF significantly reduced the IS in the MI/RI-DIL, MI/RI-LHF, MI/RI-MHF, and MI/RI-HHF groups (*p* < 0.05; Figure 2B,D).

### 2.4. HF Recovers Myocardial Architecture after MI/RI

**Figure 3:**
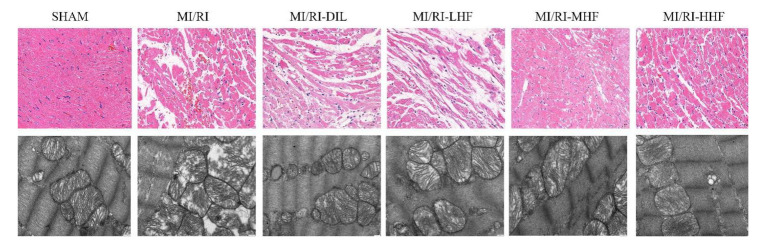
Effect of HF on the histopathological (400×, scale = 50 μm, *n* = 3) and ultrastructural changes (20,000×, scale = 1 μm, *n* = 3) of the LV from each group.

Hematoxylin-eosin (HE) staining and transmission electron microscopy (TEM) were used to detect the abnormal myocardial tissue. The SHAM group showed normal myocardial architecture. Compare with the SHAM group, the cardiomyocytes displayed degeneration and atrophy of the myocardial fibers and exudation of red blood cells in the MI/RI group. Compared with the MI/RI group, the pretreatment with DIL and HF alleviated the degeneration of the myocardial fibers (Figure 2). Based on the TEM, the SHAM group showed normal interfibrillar mitochondria (IFM) and myocardial fibers, while the MI/RI rats demonstrated injured IFMs. However, the pretreatment with DIL and HF relieved these abovementioned changes in the MI/RI-DIL, MI/RI-LHF, MI/RI-MHF, and MI/RI-HHF groups (Figure 3).

### 2.5. HF Decreases Myocardial Injury Markers after MI/RI

**Figure 4:**
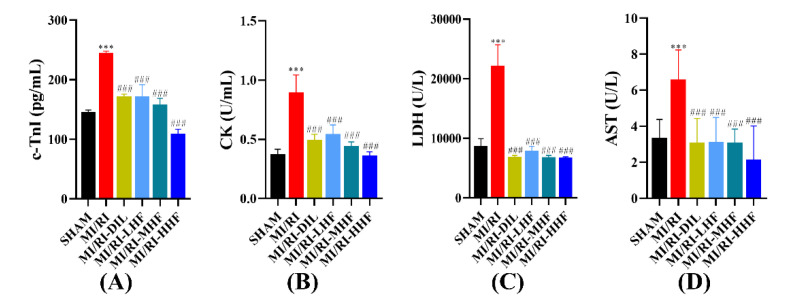
Effects of HF on myocardial injury markers. (**A**) c-TnI (*n* = 15~16). (**B**) CK (*n* = 15~16). (**C**) LDH (*n* = 15~16). (**D**) AST (*n* = 15~16). Results were expressed as mean ± SD. *** *p* < 0.001 vs. SHAM group. ^###^ *p* < 0.001 vs. MI/RI group.

Myocardial injury markers included cardiac troponin I (c-TnI), creatine kinase (CK), lactate dehydrogenase (LDH), and aspartate transaminase (AST), which were used to assess the injured cardiomyocytes. Compared with the SHAM group, an apparent increase in the serum of c-TnI, CK, LDH, and AST was observed in the MI/RI group (*p* < 0.05; Figure 4A–D). Compared with the MI/RI group, the pretreatment with DIL and HF significantly decreased the level of c-TnI, CK, LDH, and AST in the MI/RI-DIL, MI/RI-LHF, MI/RI-MHF, and MI/RI-HHF groups (*p* < 0.05; Figure 4A–D).

### 2.6. HF Inhibits the Inflammatory Cytokines after MI/RI

**Figure 5:**
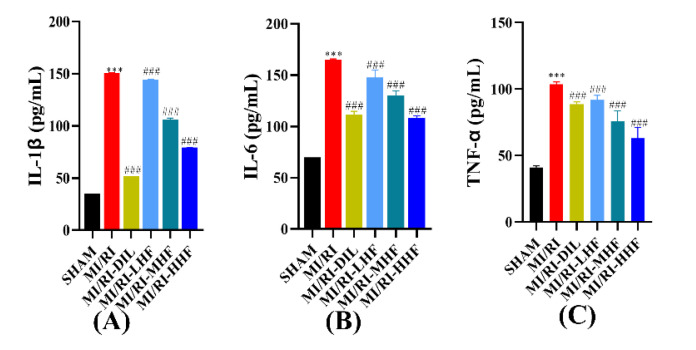
Effects of HF on inflammatory cytokines. (**A**) IL-1β(*n* = 15~16). (**B**) IL-6 (*n* = 15~16). (**C**) TNF-α (*n* = 15~16). Results were expressed as mean ± SD. *** *p* < 0.001 vs. SHAM group. ^###^ *p* < 0.001 vs. MI/RI group.

Inflammatory cytokines included IL-1β, IL-6, and TNF-α, which were used to appraise the inflammation of the cardiomyocytes. Compared with the SHAM group, an obvious increase in the serum of IL-1β, IL-6, and TNF-α was observed in the MI/RI group (*p* < 0.05; Figure 5A–C). Compared with MI/RI group, the pretreatment with DIL and HF significantly inhibited the level of IL-1β, IL-6, and TNF-α in the MI/RI-DIL, MI/RI-LHF, MI/RI-MHF, and MI/RI-HHF groups (*p* < 0.05; Figure 5A–C).

### 2.7. HF Modulates Superoxide Dismutase and Malondialdehyde after MI/RI

**Figure 6:**
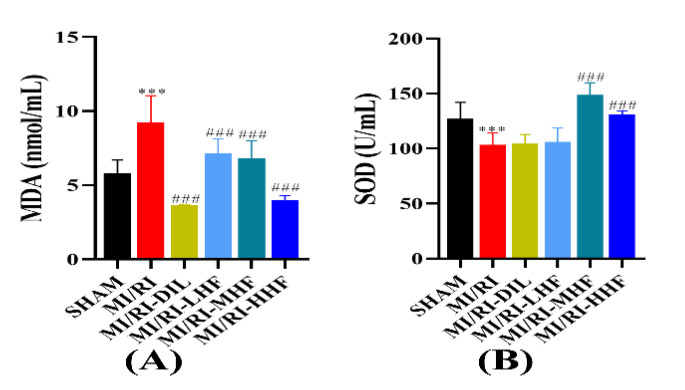
Effects of HF on MDA and SOD. (**A**) MDA(*n* = 15~16). (**B**) SOD (*n* = 15~16). Results were expressed as mean ± SD. *** *p* < 0.001 vs. SHAM group. ^###^ *p* < 0.001 vs. MI/RI group.

Oxidative stress markers were comprised of superoxide dismutase (SOD) and malondialdehyde (MDA), which were used to measure the oxidative stress response of the cardiomyocytes. Compared with the SHAM group, a marked increase in the serum of MDA, and a prominent decrease in the serum of SOD, were observed in the MI/RI group (*p* < 0.05; Figure 6A,B). Compared with the MI/RI group, the pretreatment with DIL and HF significantly alleviated these alterations in the MI/RI-DIL, MI/RI-LHF, MI/RI-MHF, and MI/RI-HHF groups (*p* < 0.05; Figure 6A,B).

### 2.8. HF Attenuates Apoptosis after MI/RI

**Figure 7:**
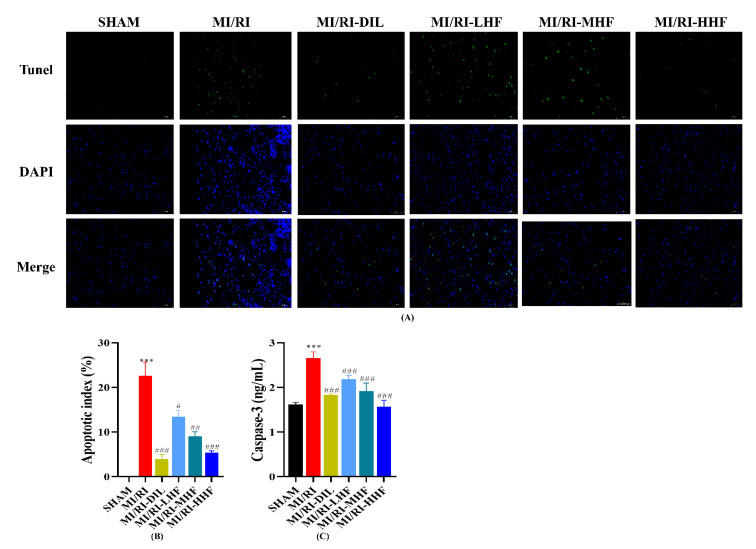
Effect of HF on TUNEL positivity and apoptotic markers. (**A**) The results of the TUNEL assay (400×). (**B**) Apoptotic index. (**C**) The serum of caspase-3. *** *p* < 0.001 vs. SHAM group. ^#^ *p* < 0.05, ^##^ *p* < 0.01, ^###^ *p* < 0.001 vs. MI/RI group.

A TdT-mediated dUTP nick end labeling (TUNEL) assay was conducted to explore DNA fragments in the nucleus of the apoptotic cells. A remarkable increase in green apoptotic cardiomyocytes was observed. In other words, TUNEL-positive cells were observed the LV of the MI/RI rats. Conversely, rare TUNEL-positive apoptotic nuclei were detected after the pretreatment with DIL and HF (Figure 7A,B). Caspase-3 was used to appraise the apoptotic cardiomyocytes. An obvious increase in the serum of caspase-3 was observed in the MI/RI group (*p* < 0.05; Figure 7C). Compared with the MI/RI group, the pretreatment with DIL and HF significantly reduced the level of caspase-3 in the MI/RI-DIL, MI/RI-LHF, MI/RI-MHF, and MI/RI-HHF groups (*p* < 0.05; Figure 7C).

### 2.9. HF Regulates the Expression of MAPK/NF-κB mRNA after MI/RI

**Figure 8:**
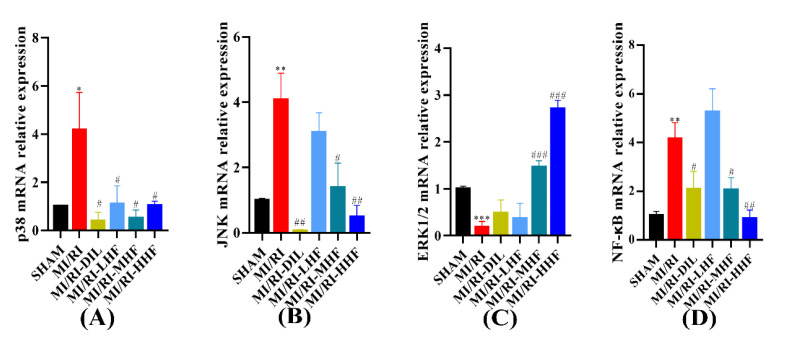
Effect of HF on MAPK/NF-κB mRNA expression. (**A**) p38 mRNA relative expression. (**B**) JNK mRNA relative expression. (**C**) ERK1/2 mRNA relative expression. (**D**) NF-κB mRNA relative expression. * *p* < 0.05, ** *p* < 0.01, *** *p* < 0.001 vs. SHAM group. ^#^ *p* < 0.05, ^##^ *p* < 0.01, ^###^ *p* < 0.001 vs. MI/RI group. (*n* = 3).

We investigated the expression of extracellular regulated protein kinases1/2, (ERK1/2), p38, c-Jun N-terminal kinase (JNK), and NF-κB at the level of mRNA, which constitute signaling pathways in the inflammatory response of the cardiomyocytes. Compared with the SHAM group, the expression of p38, JNK, and NF-κB mRNA increased in the MI/RI group, and the levels of ERK1/2 mRNA decreased in the MI/RI group. After the pretreatment with HF and DIL, the alteration tended to be in the opposite direction of the MI/RI group in the MI/RI-DIL, MI/RI-LHF, MI/RI-MHF, and MI/RI-HHF groups (*p* < 0.05; Figure 8A–D).

### 2.10. HF Modulates the Protein of the MAPK/NF-κB Signaling Pathway after MI/RI

**Figure 9:**
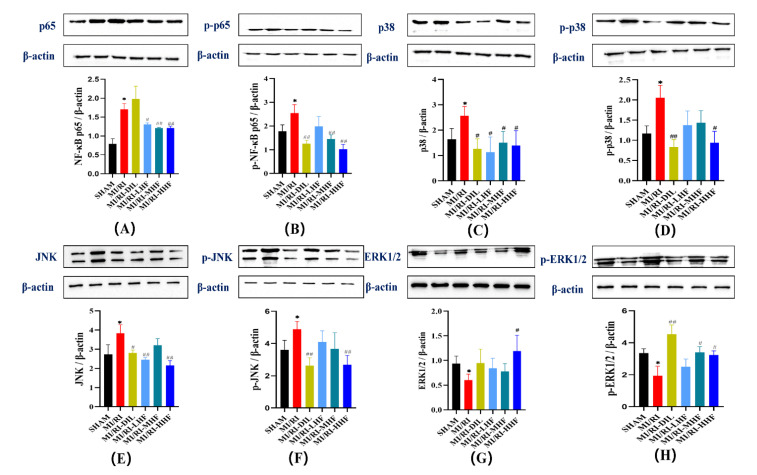
Effect of HF on MAPK/NF-κB protein expression. (**A**) NF-κB p65 protein relative expression. (**B**) p-NF-κB p65 protein relative expression. (**C**) p38 protein relative expression. (**D**) p-p38 protein relative expression. (**E**) JNK protein relative expression. (**F**) p-JNK protein relative expression. (**G**) ERK1/2 protein relative expression. (**H**) p-ERK1/2 protein relative expression. * *p* < 0.05 vs. SHAM group. ^#^ *p* < 0.05, ^##^ *p* < 0.01 vs. MI/RI group, (*n* = 3).

We also measured the expression of ERK1/2, p38, JNK, and NF-κB in terms of the levels of proteins, which constitute the signaling pathways in the inflammatory response of the cardiomyocytes. Compared with the SHAM group, there was an upregulation of NF-κB p65, p38 and JNK, phosphorylation of NF-κB p65, p38 and JNK, downregulation of ERK1/2, and phosphorylation of ERK1/2 in the MI/RI group. After the pretreatment with HF and DIL, the expression of these proteins all changed in the opposite direction of the MI/RI group in the MI/RI-DIL, MI/RI-LHF, MI/RI-MHF, and MI/RI-HHF groups (*p* < 0.05; Figure 9A–H).

### 2.11. Molecular Docking

**Figure 10:**
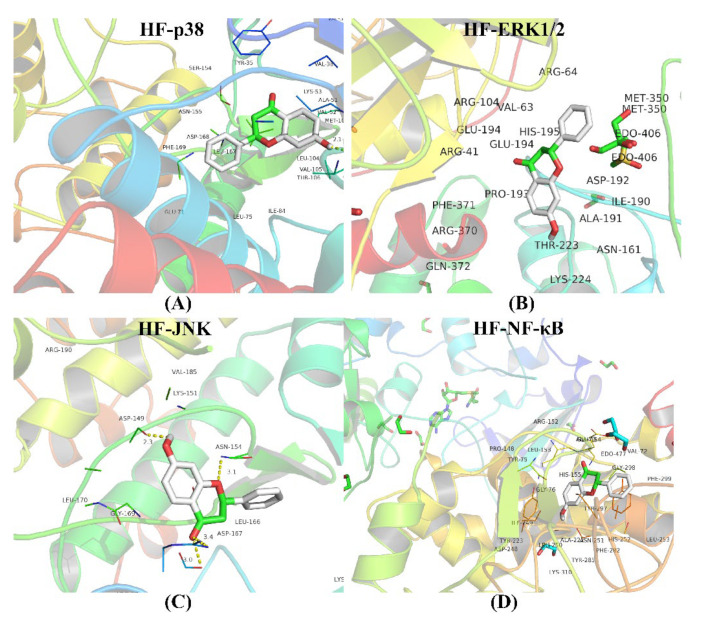
Molecular docking. (**A**) The combination between HF and p38. (**B**) The combination between HF and ERK1/2. (**C**) The combination between HF and JNK. (**D**) The combination between HF and NF-κB. The binding affinities are all less than 5 kcal/moL.

The intersection between the protein of the MAPK/NF-κB signaling pathway and HF and the target gene was investigated. The values of the binding affinity were greater than 5 kcal/moL, which indicated that the HF, p38, JNK, ERK1/2, and NF-κB had stable combinations (Figure 10). The results of the binding affinity are shown in detail (Table S2).

## 3. Discussion

**Figure 11:**
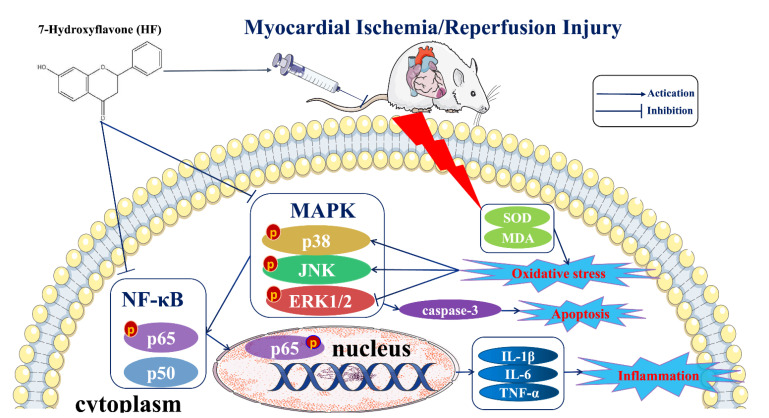
A schematic diagram summarizing the underlying mechanisms in this study. HF can protect the heart from myocardial ischemia/reperfusion injury. The potential mechanisms of this effect can be connected with the activation of the MAPK/NF-κB signaling pathway, reduction in the inflammatory cytokines (IL-1β, IL-6, and TNF-α) and apoptotic factor (caspase-3), and the enhancement of the antioxidant levels (SOD and MDA).

IHD occurring due to blocked coronary arteries has become one of the leading causes of morbidity and mortality in the world [5,18]. Reperfusion therapy is a double-edged sword that can restore the coronary blood flow to prevent the ischemic cardiomyocytes from injury but can also result in extra damage to the cardiomyocytes, which is called MI/RI [19,20]. Clinical symptoms of MI/RI include reperfusion arrhythmias, heart failure, and even sudden cardiac death [21]. Ensuring the blood supply recovery of the ischemic myocardial tissue is an important goal in the efforts to prevent or alleviate MI/RI. As an essential component of traditional Chinese medicines, TTMs have advantages in terms of their efficacy and fewer side effects due to their many targets, which make them unique in the treatment of cardiovascular disease [22,23]. In our previous study, we found that *O. falcata* extract demonstrated significant protective effects against MIRI, but the underlying mechanism is not clear. UHPLC–MS/MS analysis has been used to identify the main active component [15,24]. Eventually, HF was proven as likely to be the medicinal substance of *O. falcata* acting against MI/RI. Consequently, an LAD-induced MI/RI rat model was used to evaluate the effects of HF and its potential mechanisms. In our study, we found that HF had a protective effect on MI/RI by modulating inflammation and the MAPK/NF-κB signaling pathway (Figure 11).

HF is a kind of phenolic ingredient of *O. falcata* and has been testified to offer significant protection against cardiac or cerebral vascular diseases [25,26]. HF has been proven to efficiently protect renal cells in rats from nicotine-induced oxidative stress via the extracellular regulated protein kinases (ERK)/nuclear factor erythroid 2-related factor 2 (Nrf2)/heme oxygenase-1 (HO-1) signaling pathway [27]. Moreover, another study has demonstrated that HF can protect RAW264.7 cells by inhibiting inflammation via suppressing the production of IL-6 and TNF-α [28]. HF reduces chemotherapy-induced neuropathic pain by inhibiting the NF-κB inflammatory pathways [16]. Thus, these studies mentioned above support the conclusion that HF has the effects of inhibiting inflammation, anti-oxidation, and protecting the blood vessels. This is consistent with the results obtained in this study.

DIL, a kind of calcium channel blocker used for the treatment of coronary artery disease, was selected as a positive drug to testify to the HF-mediated effects. Consistent with previous findings [29,30,31], our results suggested that DIL attenuates myocardial injury while promoting myocardial survival. We also compared the protection against MIRI offered by HF and DIL. With some parameters, HF seemed to be more effective than DIL. Thus, on the one hand, these results showed that the positive drug we selected is acceptable. On the other hand, they also indicated that HF and DIL have similar cardioprotective effects.

IS is a significant parameter for evaluating the effectiveness of cardiovascular treatments, which can be used to assess the status and prognosis of coronary artery disease in clinics. HF and DIL reduced IS. AST, LDH, CK, and c-TnI are regarded as markers of myocardial injury, and when the cardiomyocytes are ruptured, they are released in great quantities into the bloodstream in MI/RI, triggering a series of subsequent pathological responses [32,33]. The results of our study showed a significant elevation in the serum of AST, LDH, CK, and c-TnI in the MI/RI group. However, the pretreatment with HF and DIL inhibited the increase in the markers of myocardial injury, including AST, LDH, CK, and c-TnI. In summary, these results indicated that the protective effects of HF and DIL against MI/RI might be effected by maintaining the stability of the cardiomyocyte membrane while reducing the release of the markers of myocardial injury in order to decrease the IS.

In this study, the cardiomyocytes underwent morphological and pathological changes after ligation for 45 min, causing occlusion of the LAD, and then 120 min of perfusion. The HE staining revealed a marked degeneration and atrophy of the myocardial fibers and the exudation of red blood cells. These morphological alterations were alleviated by the HF and DIL treatments. It is well known that the categories of the mitochondria include subsarcolemmal mitochondria (SSM), IFM, and perinuclear mitochondria (PNM). From the results of the TEM, IFMs were detected, which ensured that the heart carried out energetically demanding work [34]. In the context of MI/RI, the IFMs were damaged in our study. Damaged IFMs might be involved in mitophagy [34]. HF and DIL could alleviate the myocardial ultrastructural injury.

The TUNEL assay stained intact single apoptotic nuclei in situ in order to accurately reflect the characteristics of apoptosis. The results of the TUNEL assay indicated that HF and DIL could reduce apoptosis during MI/RI to decrease the extent of myocardial injury. A significant pathological alteration caused by MI/RI is apoptosis. The activation of caspase-3 is affected by B-cell lymphoma-2 (BCL2) [35,36]. In this study, the expression of caspase-3 was increased in the MI/RI group. The administration of HF and DIL could reduce the expression of caspase-3.

Oxidative stress is also a significant factor in MI/RI. MDA levels could reveal the severity of lipid peroxidation. SOD is a kind of free radical-scavenging enzyme, which acts as the first line of antioxidant stress defense by scavenging reactive oxygen radicals. In this study, the pretreatment with HF and DIL could reduce the elevation in the MDA levels and increase the activity of SOD [37]. These results indicated that HF and DIL could protect the cardiomyocytes from oxidative damage by increasing the level of endogenous antioxidant enzymes. Based on other studies, as well as our research at the cellular level, it is likely that HF exerts anti-oxidative stress effects by directly regulating the release of ROS, which in turn exerts subsequent anti-inflammatory and anti-apoptotic effects [15,38].

The excessive inflammatory response is a significant factor in the MI/RI. It has been reported that MI/RI can increasingly induce the production of IL-6 [39]. TNF-α can suppress the myocardial systolic function and provoke the production of adhesion molecules by endothelial cells and neutrophils, thus resulting in the apoptosis of the cardiomyocytes [40,41]. Taken together, the measurement of TNF-α and IL-6 concentrations could indirectly reveal the concentration of the inflammatory responses. In this study, the pretreatment with HF and DIL could reduce the concentration of IL-1β, IL-6, and TNF-α. Thus, these results showed that HF might be mediated through inflammatory responses and apoptosis, mostly via TNF-α-mediated death receptor pathway activation.

It is also well known that MI/RI can lead to the development of inflammation, oxidative stress, and apoptosis in the cardiomyocytes. In particular, p38 and JNK have been testified to play critical roles in the transmission of inflammatory and apoptotic signals [42]. The activation of the JNK signaling pathway leads to an increase in IL-6 [43]. During the state of oxidative stress, p38/JNK signaling pathways are activated and result in the translocation of B-cell lymphoma-*2*-associated X (Bax) to the mitochondrial pathway and initiating the mitochondrial-dependent apoptosis pathway. The inhibition of p38 can reduce the apoptosis of the cardiomyocytes and recover the cardiac function [44,45]. What is more, melatonin has been proven to inhibit the p38/JNK signaling pathway to ensure cardiomyocyte survival in the model of hepatic ischemia-reperfusion injury [46]. In our study, the results showed that HF could inhibit the expression of p38 and JNK, decrease the phosphorylation of JNK, and increase the expression of ERK1/2 and the phosphorylation of ERK1/2 to protect the heart from MI/RI. Molecular docking also confirmed the abovementioned results. Asiatic acid relieves MI/RI by inhibiting the release of ROS in the mitochondria-dependent apoptosis pathway [38]. Sevoflurane pre-conditioning relieved MI/RI by the antioxidant, and then by regulating the p38 and ERK signaling pathways [47]. In our previous study, we found that the flavonoids of *O. falcata* alleviated the MI/RI by inhibiting the release of ROS. Based on the abovementioned evidence, we hold the view that HF indirectly regulates the MAPK signaling pathways. Thus, it is convincing to understand the inhibition of the p38/JNK and activation of the ERK1/2 signaling pathways as mechanisms contributing to the protection of the heart from MI/RI.

NF-κB is composed of a class of transcription factors, which play a significant role in inflammation, cell proliferation, and survival [48]. NF-κB consists of p50 and p65 subunits [49]. In the context of MI/RI, NF-κB and IκB are released by NF-κB/IκB complexes. NF-κB is phosphorylated and translocated into the nucleus to trigger acute inflammation in the cardiomyocytes. It was shown that the role of NF-κB in myocardial inflammation is enacted by promoting the expression of inflammatory factors [50,51]. In our study, the effect of HF on the suppression of the NF-κB signaling pathway was evaluated. In the line with this, our results demonstrated that HF could inhibit the phosphorylation of NF-κB p65 to reduce the expression of IL-1β, IL-6, and TNF-α. Moreover, molecular docking was used to demonstrate that HF has a strong affinity with NF-κB.

## 4. Materials and Methods

### 4.1. Materials

HF (CAS: 6665-86-7; H0852; purity ≥ 97%) was purchased from TCI Shanghai Chemical Industry Development Co., Ltd. (Shanghai, China). Diltiazem was purchased from Shanghai Pharmaceutical Co., Ltd. (Lot: H10970375, Shanghai, China). TTC (Lot: G3005) was purchased from Beijing Solarbio Science & Technology Co., Ltd. (Beijing, China). AST (Lot: C010-2-1), CK (Lot: A032-1-1), LDH (Lot: A020-2), SOD (Lot: A001-3), and MDA (Lot: A003-1) were purchased from Nanjing Jiancheng Bioengineering Institute (Jiangsu, Nanjing, China). Rat c-TnI (Lot: E-EL-R1253c), IL-1β (Lot: E-EL-R0012c), IL-6 (Lot: E-EL-R0015c), TNF-α (Lot: E-EL-R2856c), and Caspase-3 (Lot: E-EL-R0160c) ELISA kits were purchased from Elabscience Biotechnology Co., Ltd. (Wuhan, China). The TUNEL assay kit (Lot: 49330900) was purchased from Roche, Switzerland. TRIzol reagent (Cat#RK145), the first-strand cDNA synthesis kit (Cat#RK145), and SYBR green supermix (Cat#RK145) were purchased from Tiangen Biotech Co., Ltd. (Beijing, China). The anti-p-p38 (Lot. WLP1576), anti-p38 (Lot. WL00764), anti-p-ERK1/2 (Lot. WLP1512), anti-ERK1/2 (Lot. WL01864), anti-p-JNK (Lot. WL01813), anti-JNK (Lot. WL01295), anti-p-NF-κB p65 (Lot. WL02169), anti-NF-κB p65 (Lot. WL01273b) and HRP-conjugated goat anti-rabbit antibody (Lot. WLA023a) were purchased from Wanlei Biotechnology Co., Ltd. (Shenyang, China).

### 4.2. Experimental Animals

All study protocols were performed and were approved by the Ethics Committee of the Medical College of Qinghai University. All endeavors were intended to reduce the population of animals sacrificed and to reduce animal suffering. Sprague Dawley (SD) male rats (*n* = 96, weight = 170 ± 10 g, 8 weeks old) were purchased from HFK Bioscience Co., Ltd. (Beijing, China). After 7 days of acclimatization, all rats were randomly divided into 6 groups: (1) sham group (SHAM), (2) MI/RI group (MI/RI), (3) MI/RI-Diltiazem (MI/RI-DIL), (4) MI/RI-low dose of HF (MI/RI-LHF), (5) MI/RI-medium dose of HF (MI/RI-MHF), and (6) MI/RI-high dose of HF (MI/RI-HHF). The SHAM and MI/RI groups were injected intraperitoneally with 0.9% saline for seven days. The MI/RI-DIL group were injected intraperitoneally with DIL (10 mg/kg body weight) for seven days [30]. The MI/RI-LHF, MI/RI-MHF, and MI/RI-HHF groups were injected intraperitoneally with HF (5, 10, and 20 mg/kg body weight, respectively) for seven days. All operations were performed 12 h after the last administration. For rats in the SHAM group, a sham operation was performed without ligating the LAD, while rats were ligated for 45 min to cause the occlusion of then LAD and then underwent 120 min of perfusion in the MI/RI, MI/RI-DIL, MI/RI-LHF, MI/RI-MHF, and MI/RI-HHF groups. BIO PAC MP150 was used to acquire the HR and ECG. After reperfusion, left ventricle (LV) samples were obtained and immediately frozen at −80 °C for further analysis. Blood samples were acquired from the abdominal aorta and centrifuged at 4 °C (3000 rpm for 15 min) to acquire samples, which were stored at −80 °C for the follow-up study.

### 4.3. Myocardial Infarct Area Measurement

The rat LVs were cut into five sections and stained in 2% TTC at 37 ℃ for 15 min in darkness [52]. The slices were put into a 10% formal-saline solution for one day. White cardiac tissues showed the heart infarct size, while red parts indicated normal tissues. Image-Pro Plus software was used to analyze the IS.

### 4.4. Morphological and Histological Analysis

The left ventricles were fixed in 4% paraformaldehyde, embedded in paraffin, and cut to a 4 μm thickness. HE was used to stain the slices for the histopathological examination. Images were obtained using 3DHISTECH (Pannoramic 250, Budapest, Hungary). All these histopathological changes were assessed by a blinded method and the observation was performed using a common optical microscope.

### 4.5. TEM

LVs were first prefixed with 3% glutaraldehyde followed by post-fixation in 1% osmium tetroxide, then dehydrated in series acetone, infiltrated for a longer period with Epox 812, and embedded. The staining of the semithin and ultrathin sections was performed using methylene blue and uranyl acetate and lead citrate, respectively. The results were determined using a transmission electron microscope (JEM-1400-FLASH, Tokyo, Japan).

### 4.6. TUNEL Assay

Apoptosis was assayed using the TUNEL assay kit according to the instructions of the manufacturer. In the statistics, the green TUNEL-positive cells were defined as apoptotic cells. The blue cells were deemed surviving cardiomyocytes. The results were observed under a fluorescence microscope at 400× magnification. All measurements were carried out blindly.

### 4.7. Markers of Myocardial Injury Test

The serum was obtained from the blood samples and centrifuged at 4 °C (3000 rpm for 15 min). The serum levels of AST, CK, and LDH were assayed following the instructions of the Nanjing Jiancheng Bioengineering Institute. The c-TnI ELISA kit was used to assay the c-TnI following the methods of Elabscience Biotechnology Co., Ltd.

### 4.8. SOD and MDA Test

The serum levels of SOD and MDA were assayed using the SOD and MDA kit, according to the method of Nanjing Jiancheng Bioengineering Institute.

### 4.9. Anti-Inflammatory Activity Test

The serum levels of IL-1β, IL-6, and TNF-α were assayed using ELISA kits, following the methods of Elabscience Biotechnology Co., Ltd.

### 4.10. Antiapoptotic Activity Test

The serum level of caspase-3 was assayed using ELISA kits, following the methods of Elabscience Biotechnology Co., Ltd.

### 4.11. Quantitative Real-Time PCR

Rat heart LV tissues were harvested with the TRIzol reagent to obtain the total RNA. According to the manufacturer’s instructions, the cDNA was produced from 2000 ng of RNA in a 20 μL reaction system using the Tiangen first-strand cDNA synthesis kit. An ABI7500 real-time PCR system (Bio-rad, CA, USA) was used to perform quantitative real-time PCR (RT-qPCR), using Tiangen SYBR green supermix according to the manufacturer’s directions. In this study, we regarded β-actin as a loading control. The information about the primer sequences can be found in Table S3. Based on the 2^−ΔΔCt^ method, and once normalized to β-actin, the relative gene expression was calculated.

### 4.12. Western Blotting

Western blotting was performed as previously reported. Total proteins of the cardiac tissues were extracted using a whole cell lysis assay kit (Wanlei bio, No. WLA019a, Shenyang, China). Protein concentrations were assayed by a BCA protein assay kit (Abbkine, Cat# KTD3001, Wuhan, China). Next, 5% non-fat milk (Yamei, No. 025B1050, Shanghai, China) combined with 1×TBST was used to block the PVDF membrane and incubated with anti-p-p38 (1:700), anti-p38 (1:750), anti-p-ERK1/2 (1:300), anti-ERK1/2 (1:500), anti-p-JNK (1:500), anti-JNK (1:1000), anti-p-NF-κB p65 (1:1000), and anti-NF-κB p65 (1:1000) antibodies overnight at 4 °C. Then, 1× TBST buffer was used to wash the PVDF membrane 3 times. Then, the washed membranes were incubated with HRP-conjugated goat anti-rabbit antibody (1:3000). After washing, an enhanced chemiluminescent (ECL) kit (Wanlei bio, No. WLA006a, Shenyang, China) was used to visualize the results. Odyssey FC was used to visualize the images.

### 4.13. Molecular Docking

Molecular docking was used to demonstrate the binding affinity of the key components to core targets. The detailed steps are described below. Firstly, ChemBioDraw (18.0) was used to draw a two-dimensional structure of HF and convert it into a three-dimensional structure. Then, the most structurally similar proteins and ligands of p38, JNK, ERK1/2, and NF-κb were acquired from the RCSB PDB online platform (https://www.rcsb.org/, accessed on 16 May 2022). What is more, PyMOL Version 1.7.x. was used to acquire the processed proteins and the original ligands by removing the water and extracting the original ligand. AutoDock Vina (Version 1.5.6, The Scripps Research Institute, La Jolla, America) was used to convert the “pdb” format of the proteins and the corresponding ligands to the “pdbqt” format. Furthermore, AutoDock Vina (Version 1.5.6) was also carried out to obtain an active center pocket. Finally, molecular docking and PyMOL Version 1.7.x. were used to acquire the binding affinities and the visualization of the results.

### 4.14. Statistical Analysis

One-way ANOVA was conducted with GraphPad Prism, version 8.3.1 (GraphPad Software Inc., San Diego, CA, USA), to compare the two groups. The IS was measured using Image-Pro Plus, version 6.0.0.260 (Media Cybernetics Inc., Rockville, MD, USA). In the analysis of the results of the WB, Image J, version 1.52v (National Institutes of Health, Rasband, USA) was used. All quantitative data were shown as mean ± standard deviation (x ± SD). A value of *p* < 0.05 was recognized as statistically significant.

## 5. Conclusions

In conclusion, this study highlights that pretreatment with HF provides cardioprotection via suppressing the inflammation and modulating the MAPK/NF-κB signaling pathway. These findings indicate that HF can be used as a novel therapeutic drug to reduce MI/RI.

## Data Availability

Datasets analyzed during the present study are available from the corresponding author upon reasonable request.

## Supplementary Materials

The following supporting information can be downloaded at: https://www.mdpi.com/article/10.3390/molecules27175371/s1, Table S1: Effect of HF and DIL on anatomic data in MI/RI rat, Table S2: Outcomes of molecular docking, Table S3: Primer information of genes used for quantitative real-time PCR.

## Sample Availability

Samples of the compounds are available from the authors.

## Abbreviations

Aspartate transaminase	AST
B-cell lymphoma-2	BCL2
B-cell lymphoma-2-associated X	Bax
Cardiac troponin I	c-TnI
Caspase-3	Casp-3
c-Jun N-terminal kinase	JNK
Creatine kinase	CK
Diltiazem	DIL
Electrocardiograph	ECG
Extracellular regulated protein kinases1/2	ERK1/2
Heart rate	HR
Hematoxylin-Eosin	HE
Heme oxygenase-1	HO-1
Interleukin-1β	IL-1β
Interleukin-6	IL-6
Infarct size	IS
Ischemic heart disease	IHD
Interfibrillar mitochondria	IFM
Left anterior descending branch of the coronary artery	LAD
Left ventricle	LV
Malondialdehyde	MDA
Mitogen-activated protein kinase	MAPK
Myocardial ischemia/reperfusion injury	MI/RI
Myocardial ischemia/reperfusion injury-diltiazem	MI/RI-DIL
Myocardial ischemia/reperfusion injury-low dose of 7-hydroxyflavone	MI/RI-LHF
Myocardial ischemia/reperfusion injury-medium dose of 7-hydroxyflavone	MI/RI-MHF
Myocardial ischemia/reperfusion injury-high dose of 7-hydroxyflavone	MI/RI-MHF
Nuclear factor erythroid 2-related factor 2	NRF2
Nuclear factor-κB	NF-κB
Oxytropis falcata Bunge	O. falcata
Perinuclear mitochondria	PNM
7-Hydroxyflavone	HF
Sprague Dawley	SD
Subsarcolemmal mitochondria	SSM
Superoxide dismutase	SOD
TdT-mediated dUTP nick end labeling	TUNEL
The ratio of heart weight to body weight	RHB
The ratio of liver weight to body weight	RLIB
The ratio of kidney weight to body weight	RKB
The ratio of spleen weight to body weight	RSB
The ratio of thymus weight to body weight	RTB
Transmission electron microscope	TEM
Tumor necrosis factor-α	TNF-α
Traditional Tibetan medicines	TTMs
2,3,5-Triphenyltetrazolium chloride	TTC

## References

[1] Heusch G. (2020). Myocardial ischaemia–reperfusion injury and cardioprotection in perspective. Nat. Rev. Cardiol..

[2] Liebman B., Schwaegler C., Foote A.T., Rao K.S., Marquis T., Aronshtam A., Bell S.P., Gogo P., LaChapelle R.R., Spees J.L. (2022). Human Growth Factor/Immunoglobulin Complexes for Treatment of Myocardial Ischemia-Reperfusion Injury. Front. Bioeng. Biotechnol..

[3] Lv T., Yan J., Lou Y., Zhang Z., Ye M., Zhou J., Luo F., Bi C., Lin H., Zhang J. (2022). Evaluation of Melatonin Therapy in Patients with Myocardial Ischemia-Reperfusion Injury: A Systematic Review and Meta-Analysis. Oxidative Med. Cell. Longev..

[4] Han J., Zhang H., Zhang Y., Zhang Z., Yu M., Wang S., Han F. (2022). Lingguizhugan decoction protects PC12 cells against Aβ (25–35)-induced oxidative stress and neuroinflammation by modulating NF-κB/MAPK signaling pathways. J. Ethnopharmacol..

[5] Zhong J., Liu P., Li S., Huang X., Zhang Q., Huang J., Guo Y., Chen M., Ruan Z., Qin C. (2020). A comparison of three-dimensional speckle tracking echocardiography parameters in predicting left ventricular remodeling. J. Healthc. Eng..

[6] Ni L., Lu Q., Tang M., Tao L., Zhao H., Zhang C., Yu Y., Wu X., Liu H., Cui R. (2022). Periplaneta americana extract ameliorates dextran sulfate sodium-induced ulcerative colitis via immunoregulatory and PI3K/AKT/NF-κB signaling pathways. Inflammopharmacology.

[7] Wijesekera T.P., Wu Z., Stephens N.P., Godula R., Lew L.K., Atkinson N.S. (2022). A non-nuclear NF-κB modulates alcohol sensitivity but not immunity. J. Neurosci..

[8] Li T., Lu H., Zhou L., Jia M., Zhang L., Wu H., Shan L. (2022). Growth factors-based platelet lysate rejuvenates skin against ageing through NF-κB signalling pathway: In vitro and in vivo mechanistic and clinical studies. Cell Prolif..

[9] Bhuiyan M.I.H., Young C.B., Jahan I., Hasan M.N., Fischer S., Azlan N.F.M., Liu M., Chattopadhyay A., Huang H., Kahle K.T. (2022). NF-κB Signaling-Mediated Activation of WNK-SPAK-NKCC1 Cascade in Worsened Stroke Outcomes of Ang II-Hypertensive Mice. Stroke.

[10] Myocardial ischemia-reperfusion injury and the influence of inflammation. https://www.sciencedirect.com/science/article/abs/pii/S1050173822000299.

[11] Zhang H., Kim H., Park B.W., Noh M., Kim Y., Park J., Park J.-H., Kim J.-J., Sim W.-S., Ban K. (2022). CU06-1004 enhances vascular integrity and improves cardiac remodeling by suppressing edema and inflammation in myocardial ischemia–reperfusion injury. Exp. Mol. Med..

[12] Zhang H., Liu Y., Cao X., Wang W., Cui X., Yang X., Wang Y., Shi J. (2021). Nrf2 Promotes Inflammation in Early Myocardial Ischemia-Reperfusion via Recruitment and Activation of Macrophages. Front. Immunol..

[13] Zhang Q., Guo Y., Zhang B., Liu H., Peng Y., Wang D., Zhang D. (2022). Identification of hub biomarkers of myocardial infarction by single-cell sequencing, bioinformatics, and machine learning. Front. Cardiovasc. Med..

[14] Li Q., Xu M., Li Z., Li T., Wang Y., Chen Q., Wang Y., Feng J., Yin X., Lu C. (2021). Maslinic Acid Attenuates Ischemia/Reperfusion Injury-Induced Myocardial Inflammation and Apoptosis by Regulating HMGB1-TLR4 Axis. Front. Cardiovasc. Med..

[15] Guo Y., Zhang B.-Y., Peng Y.-F., Chang L.C., Li Z.-Q., Zhang X.-X., Zhang D.-J. (2022). Mechanism of Action of Flavonoids of *Oxytropis falcata* on the Alleviation of Myocardial Ischemia–Reperfusion Injury. Molecules.

[16] Ullah R., Ali G., Rasheed A., Subhan F., Khan A., Halim S.A., Al-Harrasi A. (2022). The 7-Hydroxyflavone attenuates chemotherapy-induced neuropathic pain by targeting inflammatory pathway. Int. Immunopharmacol..

[17] Soliman M.S.M., Abdella A., Khidr Y.A., Hassan G.O.O., Al-Saman M.A., Elsanhoty R.M. (2021). Pharmacological Activities and Characterization of Phenolic and Flavonoid Compounds in Methanolic Extract of Euphorbia cuneata Vahl Aerial Parts. Molecules.

[18] Jamal S., Hassan J., Kichloo A., Ijaz S.H., Mahmoud M., Ajmal M., Paul T.K., Bailey B. (2021). Impact of Ischemic Heart Disease on Inpatient Outcomes of Atrial Fibrillation. Circulation.

[19] Chen Z., Wu J., Li S., Liu C., Ren Y. (2022). Inhibition of Myocardial Cell Apoptosis Is Important Mechanism for Ginsenoside in the Limitation of Myocardial Ischemia/Reperfusion Injury. Front. Pharmacol..

[20] Zhang Q., Guo Y., Zhang D. (2022). Network Pharmacology Integrated with Molecular Docking Elucidates the Mechanism of Wuwei Yuganzi San for the Treatment of Coronary Heart Disease. Nat. Prod. Commun..

[21] Zou R., Nie C., Pan S., Wang B., Hong X., Xi S., Bai J., Yu M., Liu J., Yang W. (2022). Co-administration of hydrogen and metformin exerts cardioprotective effects by inhibiting pyroptosis and fibrosis in diabetic cardiomyopathy. Free Radic. Biol. Med..

[22] Zhang X., Zhang Z., Wang P., Han Y., Liu L., Li J., Chen Y., Liu D., Wang J., Tian X. (2021). Bawei Chenxiang Wan Ameliorates Cardiac Hypertrophy by Activating AMPK/PPAR-α Signaling Pathway Improving Energy Metabolism. Front. Pharmacol..

[23] Zhao F., Bai R., Li J., Feng X., Jiao S., Wuken S., Ge F., Zhang Q., Zhou X., Tu P. (2020). Meconopsis horridula Hook. f. & Thomson extract and its alkaloid oleracein E exert cardioprotective effects against acute myocardial ischaemic injury in mice. J. Ethnopharmacol..

[24] Zhang D.J., Yuan W.T., Zhang B.Y., Hong E.K., Shi P., Yang Z.T., Zhang Y.H., Wang H.L. (2020). Analysis of chemical constituents in the extract and rat serum from the chloroform extract of Oxytropis falcata Bunge by HPLC-MS. Pak. J. Pharm. Sci..

[25] Sengupta B., Reilly S.M., Davis D.E., Harris K., Wadkins R.M., Ward D., Gholar D., Hampton C. (2015). Excited state proton transfer of natural flavonoids and their chromophores in duplex and tetraplex DNAs. J. Phys. Chem. B.

[26] Vajragupta O., Boonchoong P., Sumanont Y., Watanabe H., Wongkrajang Y., Kammasud N. (2003). Manganese-based complexes of radical scavengers as neuroprotective agents. Bioorg. Med. Chem..

[27] Sengupta B., Sahihi M., Dehkhodaei M., Kelly D., Arany I. (2017). Differential roles of 3-Hydroxyflavone and 7-Hydroxyflavone against nicotine-induced oxidative stress in rat renal proximal tubule cells. PLoS ONE.

[28] Jin Z., Yang Y.Z., Chen J.X., Tang Y.Z. (2017). Inhibition of pro-inflammatory mediators in RAW264.7 cells by 7-hydroxyflavone and 7,8-dihydroxyflavone. J. Pharm. Pharmacol..

[29] Yue R.C., Lu S.Z., Luo Y., Wang T., Liang H., Zeng J., Liu J., Hu H.X. (2019). Calpain silencing alleviates myocardial ischemia-reperfusion injury through the NLRP3/ASC/Caspase-1 axis in mice. Life Sci..

[30] Zhu L., Wei T., Gao J., Chang X., He H., Luo F., Zhou R., Ma C., Liu Y., Yan T. (2015). The cardioprotective effect of salidroside against myocardial ischemia reperfusion injury in rats by inhibiting apoptosis and inflammation. Apoptosis.

[31] Chen C., Lu W., Wu G., Lv L., Chen W., Huang L., Wu X., Xu N., Wu Y. (2017). Cardioprotective effects of combined therapy with diltiazem and superoxide dismutase on myocardial ischemia-reperfusion injury in rats. Life Sci..

[32] Luo Y., Pan Y.Z., Zeng C., Li G.L., Lei X.M., Liu Z., Zhou S.F. (2011). Altered serum creatine kinase level and cardiac function in ischemia-reperfusion injury during percutaneous coronary intervention. Med. Sci. Monit..

[33] Sabe S.A., Feng J., Sellke F.W., Abid M.R. (2022). Mechanisms and Clinical Implications of Endothelium-dependent Vasomotor Dysfunction in Coronary Microvasculature. Am. J. Physiol. Heart Circ. Physiol..

[34] Lu X., Thai P.N., Lu S., Pu J., Bers D.M. (2019). Intrafibrillar and perinuclear mitochondrial heterogeneity in adult cardiac myocytes. J. Mol. Cell. Cardiol..

[35] Guo M., Chen K., Lv Z., Shao Y., Zhang W., Zhao X., Li C. (2020). Bcl-2 mediates coelomocytes apoptosis by suppressing cytochrome c release in Vibrio splendidus challenged Apostichopus japonicus. Dev. Comp. Immunol..

[36] Zhai K.-F., Duan H., Chen Y., Khan G.J., Cao W.-G., Gao G.-Z., Shan L.-L., Wei Z.-J. (2018). Apoptosis effects of imperatorin on synoviocytes in rheumatoid arthritis through mitochondrial/caspase-mediated pathways. Food Funct..

[37] Zhai K.F., Duan H., Khan G.J., Xu H., Han F.K., Cao W.G., Gao G.Z., Shan L.L., Wei Z.J. (2018). Salicin from Alangium chinense Ameliorates Rheumatoid Arthritis by Modulating the Nrf2-HO-1-ROS Pathways. J. Agric. Food Chem..

[38] Yi C., Song M., Sun L., Si L., Yu D., Li B., Lu P., Wang W., Wang X. (2022). Asiatic Acid Alleviates Myocardial Ischemia-Reperfusion Injury by Inhibiting the ROS-Mediated Mitochondria-Dependent Apoptosis Pathway. Oxidative Med. Cell. Longev..

[39] Lyu M., Cui Y., Zhao T., Ning Z., Ren J., Jin X., Fan G., Zhu Y. (2018). Tnfrsf12a-Mediated Atherosclerosis Signaling and Inflammatory Response as a Common Protection Mechanism of Shuxuening Injection Against Both Myocardial and Cerebral Ischemia-Reperfusion Injuries. Front. Pharmacol..

[40] Zhai K.F., Duan H., Luo L., Cao W.G., Han F.K., Shan L.L., Fang X.M. (2017). Protective effects of paeonol on inflammatory response in IL-1β-induced human fibroblast-like synoviocytes and rheumatoid arthritis progression via modulating NF-κB pathway. Inflammopharmacology.

[41] Sun N., Wang H., Wang L. (2016). Protective effects of ghrelin against oxidative stress, inducible nitric oxide synthase and inflammation in a mouse model of myocardial ischemia/reperfusion injury via the HMGB1 and TLR4/NF-κB pathway. Mol. Med. Rep..

[42] Yue J., López J.M. (2020). Understanding MAPK Signaling Pathways in Apoptosis. Int. J. Mol. Sci..

[43] Li Z., Ding Y., Peng Y., Yu J., Pan C., Cai Y., Dong Q., Zhong Y., Zhu R., Yu K. (2022). Effects of IL-38 on Macrophages and Myocardial Ischemic Injury. Front. Immunol..

[44] Thomas C.J., Ng D.C., Patsikatheodorou N., Limengka Y., Lee M.W., Darby I.A., Woodman O.L., May C.N. (2011). Cardioprotection from ischaemia–reperfusion injury by a novel flavonol that reduces activation of p38 MAPK. Eur. J. Pharmacol..

[45] Guo W., Liu X., Li J., Shen Y., Zhou Z., Wang M., Xie Y., Feng X., Wang L., Wu X. (2018). Prdx1 alleviates cardiomyocyte apoptosis through ROS-activated MAPK pathway during myocardial ischemia/reperfusion injury. Int. J. Biol. Macromol..

[46] Zhou L., Zhao D., An H., Zhang H., Jiang C., Yang B. (2015). Melatonin prevents lung injury induced by hepatic ischemia–reperfusion through anti-inflammatory and anti-apoptosis effects. Int. Immunopharmacol..

[47] Xie D., Zhao J., Guo R., Jiao L., Zhang Y., Lau W.B., Lopez B., Christopher T., Gao E., Cao J. (2020). Sevoflurane Pre-conditioning Ameliorates Diabetic Myocardial Ischemia/Reperfusion Injury Via Differential Regulation of p38 and ERK. Sci. Rep..

[48] Liu J., Feng X., Li B., Sun Y., Jin T., Feng M., Ni Y., Liu M. (2022). Lactobacillus rhamnosus GR-1 Alleviates Escherichia coli-Induced Inflammation via NF-κB and MAPKs Signaling in Bovine Endometrial Epithelial Cells. Front. Cell Infect. Microbiol..

[49] Yan X., Zhao X., Zhou M., Sun Y., Xu T. (2022). IRF4b and IRF8 Negatively Regulate RLR-Mediated NF-κB Signaling by Targeting MITA for Degradation in Teleost Fish. Front. Immunol..

[50] Zeng S., Yi R., Tan F., Sun P., Cheng Q., Zhao X. (2022). Lactobacillus plantarum HFY05 Attenuates Carrageenan-Induced Thrombosis in Mice by Regulating NF-κB Pathway-Associated Inflammatory Responses. Front. Nutr..

[51] Yang Z., Yin X., Chen C., Huang S., Li X., Yan J., Sun Q. (2022). CircOAS3 Regulates Keratinocyte Proliferation and Psoriatic Inflammation by Interacting with Hsc70 via the JNK/STAT3/NF-κB Signaling Pathway. Inflammation.

[52] Alsahly M.B., Zakari M.O., Koch L.G., Britton S., Katwa L.C., Lust R.M. (2021). Influence of Intrinsic Aerobic Exercise Capacity and Sex on Cardiac Injury Following Acute Myocardial Ischemia and Reperfusion. Front. Cardiovasc. Med..

