# Supplementary material for: 7-Hydroxyflavone Alleviates Myocardial Ischemia/Reperfusion Injury in Rats by Regulating Inflammation
Source: Molecules. 2022 Aug 23;27(17):5371. doi: 10.3390/molecules27175371 (PMC9458087; doi:10.3390/molecules27175371)
Supplement: Supplementary file 1 [file molecules-27-05371-s001.zip › Table S1 Effect of HF and DIL on Anatomic data in MIRI rat.pdf]

Table S1. Effect of HF and DIL on Anatomic data in MI/RI rat ($\bar{x}$±*s*).

Parameters	SHAM	MI/RI	MI/RI-DIL	MI/RI-LHF	MI/RI-MHF	MI/RI-HHF
Numbers of rats	16	15	15	15	16	16
Initial BW (g)	174.44±3.20	174.60±2.67	175.33±3.13	174.93±3.79	174.81±3.37	175.06±2.81
Final BW (g)	260.94±11.10	265.47±14.17	252.26±13.66	263.20±10.80	264.88±17.13	273.69±20.57
HR (beats/min)	346.67±45.10	266.67±7.63*	400.00±34.64$^{\#}$	343.33±70.95	410.00±28.35$^{\#}$	314.00±15.10$^{\#}$
RHB (mg/g)	3.15±0.27	3.84±0.56*	3.81±0.4	3.91±0.35	3.45±0.21$^{\#}$	3.71±0.3$^{\#}$
RLIB (mg/g)	30.97±3.75	34.29±3.75*	33.37±3.63	35.07±1.92	31.13±3.44$^{\#}$	31.71±2.81$^{\#}$
RLUB (mg/g)	5.10±0.52	5.43±0.38	4.97±0.62$^{\#}$	5.27±0.50	5.01±0.47	5.15±0.52$^{\#}$
RKB (mg/g)	3.56±0.31	4.03±0.37*	4.16±0.38	4.24±0.31	3.95±0.39	4.09±0.59
RSB (mg/g)	1.88±0.23	2.50±0.60*	2.08±0.35$^{\#}$	1.81±0.24$^{\#}$	1.56±0.25$^{\#}$	2.09±0.30$^{\#}$
RTB (mg/g)	1.65±0.25	2.10±0.29*	1.68±0.39$^{\#}$	1.91±0.35	1.88±0.26$^{\#}$	1.77±0.24$^{\#}$

Results were expressed as mean ± SD. Abbreviations used as are body weight (BW), heart rate (HR), the ratio of heart weight to body weight (RHB), the ratio of liver weight to body weight (RLIB), the ratio of lung weight to body weight (RLUB), the ratio of kidney weight to body weight (RKB), the ratio of spleen weight to body weight (RSB), the ratio of thymus weight to body weight (RTB). * $p<0.05$ vs. SHAM group. $^{\#}p<0.05$ vs. MI/RI group.
