# Supplementary material for: 7-Hydroxyflavone Alleviates Myocardial Ischemia/Reperfusion Injury in Rats by Regulating Inflammation
Source: Molecules. 2022 Aug 23;27(17):5371. doi: 10.3390/molecules27175371 (PMC9458087; doi:10.3390/molecules27175371)
Supplement: Supplementary file 1 [file molecules-27-05371-s001.zip › Table S2 Outcomes of molecular docking.pdf]

**Table S2.** Outcomes of molecular docking.

| Ingredients | Targets | Affinity (kcal/mol) | Distance from best mode | |
|---|---|---|---|---|
| | | | rmsd l.b. | rmsd u.b. |
| HF | p38 | -8.3 | 0 | 0 |
| | ERK1/2 | -5.6 | 0 | 0 |
| | JNK | -5.0 | 0 | 0 |
| | NK-κB | -9.0 | 0 | 0 |
