# Supplementary material for: 7-Hydroxyflavone Alleviates Myocardial Ischemia/Reperfusion Injury in Rats by Regulating Inflammation
Source: Molecules. 2022 Aug 23;27(17):5371. doi: 10.3390/molecules27175371 (PMC9458087; doi:10.3390/molecules27175371)
Supplement: Supplementary file 1 [file molecules-27-05371-s001.zip › Table S3 Primer information of genes used for quantitative real.pdf]

Table S3. Primer information of genes used for quantitative real-time PCR.

Gene name	Primer	Primers sequences 5'-3'	Length (bp)	Temp. (°C)
NF-κB p65	Forward	TACTTGCCAGACACAGAC	18	55.02
	Reverse	TGATGCTCTTGAAGGTCTC	19	55.41
p38	Forward	GCCTGTTGCTGACCCTTATGACC	23	63.73
	Reverse	GGGGTGGTGGCACAAAGCTGAT	22	63.8
ERK1/2	Forward	GAACTCCAAGGGCTATACCAAGT	23	50.17
	Reverse	GGAGGGCAGAGACTGTAGGTAGT	23	63.73
JNK1	Forward	GTGGGGTATGCCCAAGAGG	19	61.88
	Reverse	GCCATAAAGCCCAGATAGAGC	21	59.97
β-Actin	Forward	GGAGATTACTGCCCTGGCTCCTA	23	63.18
	Reverse	GACTCATCGTACTCCTGCTTGCTG	24	63.25
